# The association of technology acceptance and physical activity on frailty in older adults during the COVID-19 pandemic period

**DOI:** 10.1186/s11556-023-00334-3

**Published:** 2023-12-19

**Authors:** Rick Yiu Cho Kwan, Joanna Wing Yan Yeung, Janet Lok Chun Lee, Vivian W. Q. Lou

**Affiliations:** 1 School of Nursing, Tung Wah College, Hong Kong, SAR China; 2 The Nethersole School of Nursing, The Chinese University of Hong Kong, Hong Kong, SAR China; 3 Department of Rehabilitation Sciences, The Hong Kong Polytechnic University, Hong Kong, SAR China; 4 Department of Social Work & Social Administration, The University of Hong Kong, Hong Kong, SAR China

**Keywords:** Frailty, Physical activity, Technology acceptance, Older adults

## Abstract

**Background:**

Physical activity was known to be the protective factor against frailty. Technology acceptance is associated with behavioural intention to technology usage. Technology has been effective in promoting healthy behaviour of physical activity. The purposes of this study were to examine the association between physical activity and technology acceptance with frailty and examine the moderation effect of technology acceptance on physical activity and frailty. We hypothesize that 1) physical activity and technology acceptance are associated with frailty, and 2) technology acceptance moderates the association of physical activity with frailty.

**Methods:**

This study employed a cross-sectional design and was conducted in the community settings of Hong Kong in 2021. Eligible participants were old people aged ≥60 and were community-dwelling. Key variables included physical activity measured by Rapid Assessment of Physical Activity (RAPA), social network measured by Lubben Social Network Scale-Six items (LSNS-6); depressive symptoms measured by Patient Health Questionnaire-Nine items (PHQ-9), technology acceptance measured by Senior Technology Acceptance Model-14 items (STAM-14) and frailty measured by Fatigue, Resistance, Ambulation, Illnesses, & Loss of Weight scale (FRAIL). Ordinal logistic regression was employed to test the hypotheses. The moderation effect was examined by introducing an interaction term formed by the multiplication of an independent variable (i.e., physical activity) and a moderating variable (i.e., technology acceptance).

**Results:**

This study recruited 380 eligible participants with a mean age of 66.5 years. Technology acceptance (Beta = − 0.031, *p* < 0.001, Pseudo-*R*^2^ = 0.087) and physical activity (Beta = − 0.182, *p* = 0.003, Pseudo-*R*^2^ = 0.027) were associated with frailty in the unadjusted models. Technology acceptance (Beta = − 0.066, *p* < 0.001) and physical activity (Beta = − 1.192, *p* < 0.001) were also associated with frailty in the fully adjusted model (Pseudo-*R*^2^ = 0.352). Interaction term formed by the multiplication of technology acceptance and physical activity (Beta = 0.012, *p* = 0.001) was associated with frailty. Physical activity was significantly associated with frailty in the lower technology acceptance subgroup (Beta = − 0.313, *p* = 0.002) in the subgroup analysis. However, in the subgroup of higher technology acceptance, the association of physical activity (Beta = 0.104, *p* = 408) on frailty became positive but not significant.

**Conclusions:**

This study showed that physical activity and technology acceptance were associated with frailty, and technology acceptance moderated the association of physical activity with frailty. This study recommends engaging older adults in physical activity to combat frailty preferentially in those with a lower level of technology acceptance.

## Introduction

Frailty is defined as a clinical syndrome with multiple causes that is characterized by diminished strength, endurance and reduced physiologic function that increases an individual’s vulnerability to developing increased dependency and/or death [1]. The prevalence of frailty among community-dwelling older adults is different around the world, 10.7% globally [2], 7% in China [3], and 16.6% in Hong Kong S.A.R [4]. Frailty has been linked with poor health outcomes, such as falls and disability, and an increased demand for health care resources [5, 6]. It has also been associated with a substantial increase in negative consequences (e.g., mortality and delirium) in COVID-19 patients [7]. The pooled prevalence of frailty among COVID-19 patients in a meta-analysis was estimated to be 45% [8], which is much higher than a recent meta-analysis which reported an estimated worldwide frailty prevalence of 18% [9]. Frailty is an important at-risk state to be managed in older adults, particularly during the COVID-19 pandemic.

Physical activity has been shown to protect against frailty in older adults [10, 11]. Physical activity is an important factor because it is modifiable and has been found to be significantly associated with frailty in older adults according to a systematic review, though the magnitude of the effect varies across contexts (e.g., types of physical activities) [12]. However, the overwhelming majority of older adults do not meet the minimum physical activity levels needed, according to a study in the United Kingdom [10]. The situation of decreased physical activity worsened under the restricted social distancing measures during the COVID pandemic [13], though it can lower the exposure risk. In Hong Kong, the social distancing measures included the closure of sports and fitness centres, restrictions on the catering business and cessation of mass events [14]. A study (*n* = 13,503) conducted in 14 countries found that social restrictions significantly reduced physical activity in older adults. In Japan (*n* = 774), a similar result was obtained. Approximately half of community-dwelling older adults reported declining physical and cognitive fitness during COVID-19 [15]. Evidence showed that decreased physical activity or limitations were also associated with frailty during the COVID-19 pandemic [16, 17]. Therefore, decreased physical activity is a key factor associated with frailty and this situation was further worsened by COVID-19. Nevertheless, frailty is also known to be associated with many factors which may be confounded with the association between physical activity and frailty (e.g., age, gender, education level, financial status, depression, falls, and social network) [18–22]. Identification of effective and modifiable factors associated with decreased physical activity and frailty after considering the effects of confounding factors is essential to develop new interventions to treat frailty.

During the COVID-19 pandemic, digital health interventions were reported to be potentially useful in promoting physical activity in older adults and improving their frailty through home-based interventions and smartphone apps [23]. E-health is also widely used in interventions for promoting physical activity and lifestyle among older adults, such as daily physical activity monitoring in an objective manner, with step counts and time spent [24, 25]. Older adults were informed and connected with the updated preventive behaviours such as social distancing measures and hand washing techniques [26]. 63% of older adults would use technology to communicate with others [27]. Technology acceptance (e.g., perceived usefulness, perceived ease of use) is associated with the behavioural intention of older adults to use technology [28]. Behavioural intention to use is associated with actual usage of technology [29]. As a result, acceptance of technology may play an important role in influencing people’s behaviors toward physical activity, which is known to be associated with frailty.

In the literature, frailty is known to be negatively associated with technology use (e.g., information and communication technologies) [30], but the association between technology acceptance and frailty is unknown. According to a systematic review, physical inactivity is associated with frailty [31]. Given the hypothetical relationships discussed above, empirical evidence demonstrating the relationships between technology acceptance, physical activity, and frailty is lacking. Therefore, we conducted a cross-sectional study to investigate the association between physical activity, technology acceptance and frailty among older adults.

### Objectives

This study aims to:

1. Examine the association between physical activity and technology acceptance with frailty, and
2. Examine the moderation effect of technology acceptance on physical activity and frailty.

We hypothesize that 1) a higher level of physical activity and a higher level of technology acceptance are associated with a lower level of frailty, and 2) technology acceptance moderates the association of physical activity with frailty.

## Methods

### Study design

This study employed a cross-sectional design. To ensure clarity of reporting, we followed the Strengthening the Reporting of Observational Studies in Epidemiology (STROBE) guidelines [32].

### Setting

The study was conducted in the community settings of Hong Kong. Data collection was conducted from September 3 2021 to November 29 2021. During this period, Hong Kong was experiencing the COVID-19 pandemic, with a mean daily number of 3.8 new confirmed cases (range = 0–31, median = 3), cumulative 12,430 cases as of November 292,021 [33], in a city with a population of approximately 7.5 million [34]. During this period, many infection control measures and social distancing policies (e.g., wearing a mask in outdoor settings, limiting the number of people co-dinning in one table) were in force [14].

### Participants

We adopted a snowball sampling method to invite eligible participants to join the study. We first recruited participants at the Institute of Active Ageing, The Hong Kong Polytechnic University and the Centre of Positive Ageing, The University of Hong Kong. Members of these organizations are aged over 50 years. They regularly participated in a variety of activities organized by the organizations, including community services, training courses, volunteer activities, and research. Subsequently, they invited their friends who were eligible to participate in the survey.

#### Inclusion criteria

1. Old adults aged ≥60 years, and
2. Community-dwelling, which is defined as living in their own homes in the past 6 months before the day of participation in the survey.

#### Exclusion criteria

1. Diagnosed dementia, self-reported

### Variables

Demographic variables included age, gender, education level, financial status, living condition, history of COVID-19 infection, and severity of COVID-19 in the living area. Physical variables included physical activity and frailty. Psychosocial variables included social network, technology acceptance, and depressive symptoms.

### Measurement

An online survey was used to collect data. The online survey was launched on Qualtrics [35], which normally takes 30 minutes to complete. Qualtrics automatically logged each response from the participants. However, this study counted the participants to be valid only if they clicked the “submit” button on the last page to indicate that they had completed the study. The invited participants received a designated hyperlink from the research team. The participants clicked on the link to complete the online survey.

#### Dependent variable

Frailty was measured by using the Fatigue, Resistance, Ambulation, Illnesses, & Loss of Weight scale (FRAIL). FRAIL comprises five dichotomous items. A total score ranges from 0 to 5. A higher score indicates a higher level of frailty. FRAIL categorizes frailty in three levels by severity (i.e., robust = 0, pre-frail = 1–2, frail = 4–5) [36]. It shares similarities with the Fried Frailty Phenotype [37], and is a screening tool for frailty. Nonetheless, according to a systematic review, FRAIL is a tool that can effectively identify frailty/prefrailty status and quantify frailty status in a graded manner in relation to mortality risk (frailty vs robustness: pooled HR = 3.53, prefrailty vs robustness: polled HR = 1.75) [38]. The Chinese FRAIL scale showed good criterion validity compared with Fried frailty phenotype (area under the curve = 0.91) and good test-retest reliability (ICC = 0.708) [39].

#### Independent variables

Physical activity was measured by the Rapid Assessment of Physical Activity (RAPA) [40]. RAPA comprises nine dichotomous items (1 = yes, 0 = no). The RAPA classifies physical activity into seven levels by intensity (from 1 = sedentary to 7 = regularly active). A higher score indicates a higher level of physical activity. WHO recommended that healthy older adults should do at least 150–300 minutes of moderate-intensity physical activity or at least 75–150 minutes of vigorous-intensity physical activity, or an equivalent combination of moderate- and vigorous-intensity activity throughout the week [41]. RAPA score below 6 (i.e., “I do 30 minutes or more a day of moderate physical activities, 5 or more days a week”) is interpreted as being physically underactive. It has been validated in older adults that it shows good validity with a good correlation with Community Health Activities Model Program for Seniors (CHAMPS) (*r* = 0.54) [40].

Technology acceptance was measured by the Senior Technology Acceptance Model-14 items (STAM-14) [42]. STAM-14 comprises 14 items. Each item is rated on a 10-point scale (from 1 = “very unsatisfied” to 10 = “very satisfied”). STAM-14 consists of four constructs: 1) attitudinal beliefs, 2) control beliefs, 3) gerontechnology anxiety, and 4) health. A total score ranges from 14 to 140. A higher score indicates a higher level of acceptance of technology. This Chinese STAM-14 scale was validated by 1012 community-dwelling individuals 55 and older in Hong Kong with good internal consistency (Cronbach’s alpha = 0.817–0.915) and construct validity (AVE = 0.455–0.795) [42].

#### Covariates

Covariates included age (years), number of falls, gender (1 = male, 2 = female), education level (1 = tertiary, 2 = secondary, and 3 = primary), financial status (a 5-point scale from 1= “much more than enough” to 5= “much less than enough”), co-living status (1 = “live alone”, 2= “live with partner/spouse”, and 3= “live with family/friend”), history of COVID-19 (1 = yes, 2 = no), and severity of COVID-19 in the living area (a 4-point scale from 1 = “not severe” to 4 = “severe”).

Social network was measured by using the Lubben Social Network Scale-Six items (LSNS-6) [43]. LSNS-6 comprises six questions with two constructs of social network sources: family and friends. Each construct consists of three items which measure the size, private conversation and help towards the social network source. Each question is rated on a 6-point scale with a total score ranging from 6 to 36. A high score indicates a higher level of social network. The Chinese version of LSNS-6 was validated and was found to be reliable and valid in predicting late-life suicidality [43] with sufficient internal consistency (Cronbach’s α = 0.832) and the Cronbach’s α were 0.90 and 0.95 for family subscale and friends subscale, respectively.

Depressive symptoms were measured by the Patient Health Questionnaire–Nine items (PHQ-9) [44]. PHQ-9 includes nine items. Each item is measured by a 4-point scale, with a total score ranging from 0 to 27. A higher score indicates a higher level of depressive symptomatology. The Chinese version of the PHQ9 was validated by comparing its scores with the clinical diagnosis of a major depressive episode using the DSM-IV criteria (AUC = 0.95, sensitivity = 0.88, specificity = 0.88) at the cut-off point of 9/10 with good internal consistency (Cronbach’s α = 0.89). The PHQ9 was validated among Chinese older adults aged equal to or over 60 years and found to have good validity (sensitivity = 0.86, specificity = 0.77) for identifying major depression in late life at the cut-off point of 9/10 [45].

### Study size

The minimum sample size required for conducting a regression analysis is 104, in addition to the number of predictors in the regression [46]. The association of technology acceptance on frailty and the moderation effect of technology acceptance on physical activity and frailty were never reported in the literature. A meta-analysis of systematic reviews revealed that the relationship between physical activity and frailty is slightly larger than small (i.e., Hedges *g* = 0.24) [12]. We estimated the sample size according to the effect size of physical activity on frailty. G*Power with the statistical test of linear multiple regression: Fixed model, *R*^2^ deviation from zero was used [47]. We assumed that the effect size is slightly larger than small (i.e., *f*^2^ = 0.03) [48], α was 0.05, power was 0.08, and the number of predictors was 2 (i.e., physical activity and technology acceptance). The total sample needed was estimated to be 325.

### Statistical methods

IBM SPSS Statistics version 26 was used for statistical analysis [49]. All the demographic variables and clinical variables were described using mean with standard deviation or frequency with the percentage depending on their levels of measurement. To test the hypotheses set in the objectives, ordinal logistic regression was employed. The dependent variable was frailty. The independent variables were physical activity and technology acceptance (i.e., objective #1). The moderation effect (i.e., objective #2) was examined by introducing an interaction term formed by the multiplication of an independent variable (i.e., physical activity) and a moderating variable (i.e., technology acceptance) [50]. Demographic [2] (i.e., age, gender) and related clinical variables [4, 51, 52] (i.e., social network and depression) were included as confounding variables because they are known to be associated with frailty in the literature. Models unadjusted and adjusted for confounders were computed separately. Psuedo-*R*-squared values were computed for unadjusted and adjusted models separately to show the change in model fitness after adding independent variables to the models. A subgroup analysis was conducted to show the moderation effect direction of technology acceptance on the association of physical activity with frailty. Two subgroups (i.e., high technology acceptance and low technology acceptance) were formed and cut off by the median score. The level of significance was set at 0.05. Missing data were replaced by the mean when the missing rate of variables was inconsequential (i.e., 5% or less).

## Results

### Participants

**Fig. 1:**
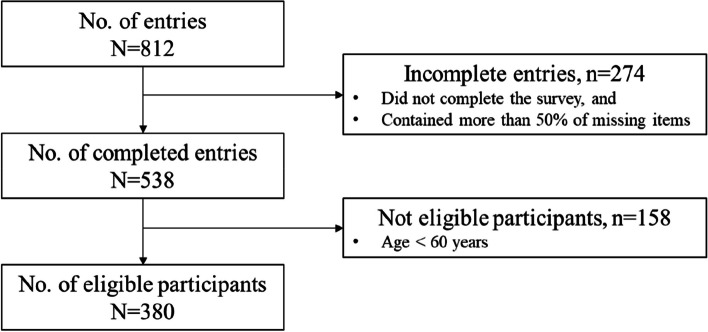
Participant flowchart

As shown in Fig. 1, 812 older adults attempted the online survey. 274 entries were invalid because the participants filled fewer than half of the total items of the survey and did not click the “submit” button to indicate that they completed the survey. Of the 538 valid inputs, there were 158 inputs not eligible because the age of the participants was lower than 60 years. Finally, there were 380 eligible and valid participants. All participants had a negligible number of missing data (i.e., < 5%), and the missing values were replaced by the mean of the variables.

### Descriptive data

As shown in Table 1, the participants’ mean age was 66.5 (SD = 5.5) years. The mean number of falls in the last 12 months was 0.59 (SD = 1.18). The majority of the participants were female (*n* = 218, 57.4%), completed secondary school (*n* = 142, 37.4%), indicated to have enough financial status (*n* = 194, 51.1%), were living with a partner/spouse (*n* = 198, 52.1%), had not been infected with COVID-19 (*n* = 379, 99.7%), and resided in an area where COVID-19 pandemic was not severe (*n* = 232, 62.1%).

**Table 1:** Participants’ demographic and clinical profile

Variables (*N* = 380)	Mean (SD) / *n* (%)
*Dependent variable*	
Frailty (FRAIL, possible range 0–5)	0 (1)^a^
0	249 (65.5)
1	78 (20.5)
2	36 (9.5)
3	16 (4.2)
4	1 (0.3)
Frailty classification	
Robust	249 (65.5)
Pre-frail	114 (30.0)
Frail	17 (4.5)
*Independent variables*	
Physical activity (RAPA, possible range: 0–7)	4.7 (1.7)
Technology acceptance (STAM-14, possible range 14–140]	94.8 (19.8)
*Covariates*	
Age in years	66.5 (5.5)
No. of falls in the last 12 months	0.59 (1.18)
Gender	
Male	162 (42.6)
Female	218 (57.4)
Educational level	
Tertiary	127 (33.4)
Secondary	142 (37.4)
Primary	111 (29.2)
Financial status	
Much more than enough	12 (3.2)
More than enough	119 (31.3)
Enough	194 (51.1)
Not enough	50 (13.2)
Much less than enough	5 (1.3)
Co-living status	
Live alone	56 (14.7)
Live with partner/spouse	198 (52.1)
Live with family	126 (33.2)
History of COVID-19	
Yes	1 (0.3)
No	379 (99.7)
Severity of COVID-19 in the living area	
Not severe	232 (62.1)
Mild	120 (31.6)
Moderate	22 (5.8)
Severe	6 (1.6)
Social network (LSNS-6, possible range 0-30)	11.9 (5.6)
Depressive symptoms (PHQ-9, possible range 0–27)	3.4 (3.5)

*COVD-19* Coronavirus Disease of 2019, *RAPA* Rapid Assessment of Physical Activity scale, *LSNS-6* Lubben Social Network Scale-Six items scale, *PHQ-9* Patient Health Questionnaire-Nine items scale, *STEAM-14* Senior Technology Acceptance Model-14 items scale, *FRAIL* Fatigue, Resistance, Ambulation, Illnesses, and loss of Weight scale

^a^The values represent a median (inter-quartile range)

The mean RAPA score (i.e., physical activity) was 4.7 (SD = 1.7), mean LSNS-6 score (i.e., social network) was 11.9 (SD = 5.6), mean PHQ-9 score (i.e., depression symptoms) was 3.4 (SD = 3.5), mean STAM-14 score (i.e., technology acceptance) was 94.8 (SD = 19.8), and the median FRAIL score (i.e., frailty) was 0 (IQR = 1). The majority of them were classified as robust (*n* = 249, 65.5%).

### Main results

#### Objective #1

As shown in Table 2, technology acceptance (Beta = − 0.031, *p* < 0.001, Pseudo-*R*^2^ = 0.087) and physical activity (Beta = − 0.182, *p* = 0.003, Pseudo-*R*^2^ = 0.027) were associated with frailty in the unadjusted models. Technology acceptance (Beta = − 0.067, *p* < 0.001), physical activity (Beta = − 0.895, *p* = 0.002), and the interaction term of [technology acceptance]*[physical activity] (Beta = 0.009, *p* = 0.005) were associated with frailty with better fitness (Pseudo-*R*^2^ = 0.116) in the adjusted model including factors of technology acceptance, physical activity, and technology acceptance (Beta = − 0.066, *p* < 0.001), physical activity (Beta = − 1.192, *p* < 0.001) and the interaction term of [technology acceptance]*[physical activity] (Beta = 0.012, *p* = 0.001) remained associated with frailty with even better fitness which better explains the phenomena (Pseudo-*R*^2^ = 0.352) in the further adjusted model.

**Table 2:** Effects of phyical activity and technology acceptance on frailty

*Dependent variable*	Unadjusted models			Adjusted model Pseudo-*R*^2^ = 0.116		Further adjusted model^#^Pseudo-*R*^2^ = 0.352	
Frailty (FRAIL)	Beta (SE)	*p*-values	Pseudo-*R*^2^	Beta (SE)	*p*-values	Beta (SE)	*p*-values
*Independent variables*							
Technology acceptance (STAM)	−0.031 (0.006)	< 0.001*	0.087	−0.067 (0.015)	< 0.001*	− 0.066 (0.017)	< 0.001*
Physical activity (RAPA)	−0.182 (0.061)	0.003*	0.027	−0.895 (0.287)	0.002*	−1.192 (0.325)	< 0.001*
[Technology acceptance] * [Physical activity]				0.009 (0.003)	0.005*	0.012 (0.004)	0.001*

*p < 0.05, *FRAIL* Fatigue, Resistance, Ambulation, Illnesses, & Loss of Weight scale, *STAM* Senior Technology Acceptance Model, *RAPA* Rapid Assessment of Physical Activity, *COVD-19* Coronavirus Disease of 2019, *PHQ-9* Patient Health Questionnaire-nine items, *LSNS-6* Lubben Social Network Scale-six items

# The model adjusted for covariates including age, gender, education levels, financial status, co-living status, no. of falls in the last 12 months, COVID-19 severity, depression, and social network

#### Objective #2

As shown in Table 2, the interaction term formed by the multiplication of technology acceptance and physical activity (Beta = 0.009, *p* = 0.005) was associated with frailty (Column 2). The interaction term (Beta = 0.012, *p* = 0.001) remained associated with frailty in the fully adjusted model (Column 3).

As shown in Table 3, in the subgroup analysis, the association of physical activity (Beta = − 0.313, *p* = 0.002) with frailty in the subgroup of lower technology acceptance was significantly negative. However, in the subgroup of higher technology acceptance, the relationship between physical activity and frailty (Beta = 0.104, *p* = 0.408) became positive but not significant.

**Table 3:** Comparing the effects of physical activity on frailty between subgroups of lower and higher technology acceptance

*Dependent variable*	Subgroup 1 (*N* = 195)^a^ Lower technology acceptance STAM score ≤ 96 (median)		Subgroup 2 (*N* = 185)^a^ Higher technology acceptance STAM score > 96 (median)	
Frailty (FRAIL)	Beta (SE)	*p*-values	Beta (SE)	*p*-values
*Independent variables*				
Physical activity (RAPA)	−0.313 (0.099)	0.002*	0.104 (0.126)	0.408

*p-value< 0.05, *STAM* Senior Technology Acceptance Model, *RAPA* Rapid Assessment of Physical Activity

^a^The model adjusted for covariates including age, gender, education levels, financial status, co-living status, no. of falls in the last 12 months, COVID-19 severity, depression, and social network

## Discussion

**Fig. 2:**
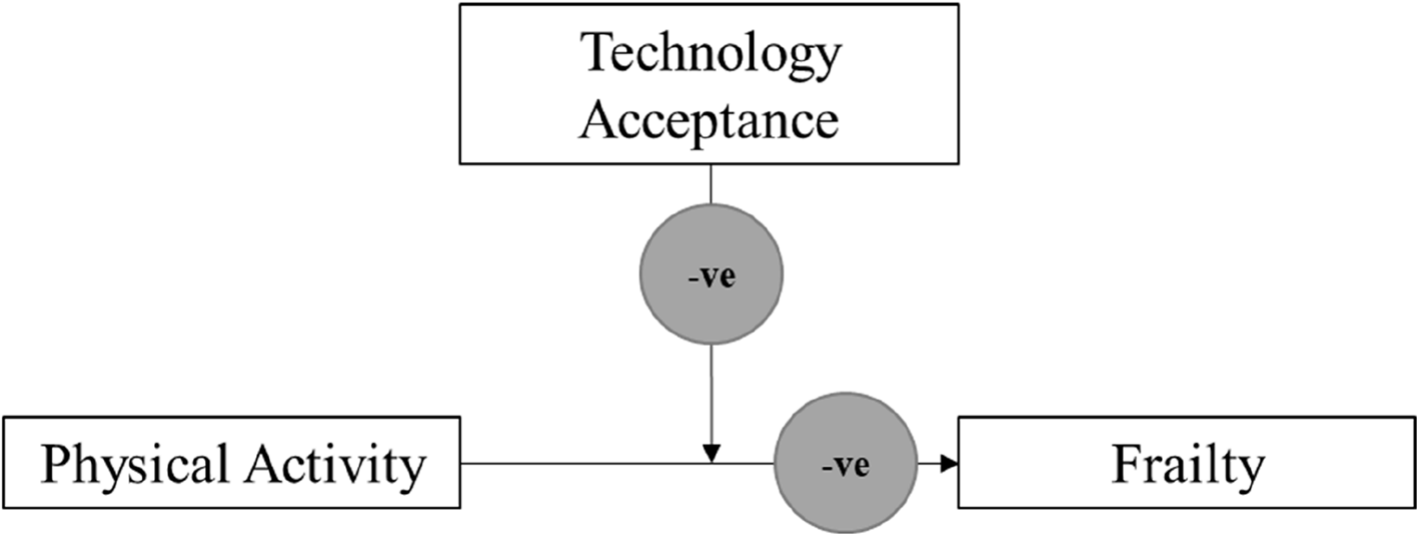
Moderation effect of technology acceptance on the effect of physical activity on frailty

To the best of our knowledge, this is the first study to report the associations between physical activity, technology acceptance and frailty among older adults. There are three key findings which support the hypotheses. First, physical activity is associated with frailty. Second, technology acceptance may be protective against frailty. Third, technology acceptance negatively moderates the association of physical activity with frailty (as shown in Fig. 2). Physical activity’s protective effect on frailty diminishes in people with a higher level of technology acceptance. These findings have a number of ramifications.

Physical activity can lower frailty among older adults is well documented in literature [10, 11]. This study yielded a similar. Under the COVID-19 pandemic, because of quarantine and social distancing measures, the level of participation in physical activity of community-dwelling older adults was reported to decline, albeit the fact that they expressed the need to remain physically active [53, 54]. The decline of participation in physical activity imposes the risk of older adults developing frailty, as the findings of this study once again reinforced this risk. Engaging older adults in an adequate amount of physical activity during the COVID-19 pandemic has become a priority public health agenda.

According to the findings of this study, technology acceptance is directly related to frailty. This finding is consistent with a recent study that found frailer people have a lower level of technology acceptance [55]. Recently, many m-health technologies have facilitated older adults with frailty to participate in physical activity in in-door settings and communities close to their homes [56, 57]. These technology-enhanced physical activity interventions are effective and might also be suitable in the context of COVID-19. This study reinforces the use of these technology-enhanced interventions to sustain the participation in physical activity of community-dwelling older adults to prevent the development of frailty. During the COVID-19 pandemic, governments of many countries invested much more in the use of new technologies for the continuation of activities [58]. Further study should also examine if these endeavours have improved technology acceptance in older adults, as well as whether the promotion of technology acceptance may also lead to a reduction of frailty. It could be a novel health promotion policy in the post-COVID era.

One possible explanation is that people with higher levels of technology acceptance may use technology more to engage in more physical activity during COVID-19. According to a systematic review, technology acceptance, particularly behavioral intention to use, is likely to be related to actual usage [29]. Technology usage and technical ability are associated with a higher level of physical activity [59]. For example, older adults use wearable sensors to engage themselves in a higher level of physical activity [20, 24]. According to the evidence, compliance with technology-based exercise is good and can provide a long-term promotion of physical activities [60]. Furthermore, even during COVID-19, when quarantine and social distancing policies were in effect, and older adults’ life-space mobility was limited within their residing areas, the physical activity level of some older adults remained satisfactory, and it was found to be associated with a lower level of sarcopenia [61]. According to the literature, attitude towards technology or actual technological use plays a role in community-dwelling older adults’ physical activity behaviour, which is protective against frailty. This study suggests that promoting technology acceptance among older adults is possibly a public health strategy to combat frailty in the community-dwelling older adults, even during a pandemic when social distancing and infection control policies are in force. Future studies should devise interventional studies to confirm this association.

Another key finding of this study was that technology acceptance negatively moderated the association of physical activity with frailty in older adults. In other words, the protective effect of physical activity on frailty is only prominent among those with a lower level of technology acceptance. The size of the effect of physical activities on frailty in older adults varied across contexts in the literature [12]. This study identified a novel factor moderating the effect of physical activity on frailty in older adults. This contrasted with the findings in the literature that a positive dose-response relationship usually exists on the effect of physical activity on frailty in older adults [62]. The reason could be that the dose-response relationship between physical activity and frailty in people who are frail or robust could be different. The sample of this study is generally less frail, and people who are less frail in this sample are mostly robust. This study showed that frailty is lower in people with a higher level of technology acceptance. These findings may suggest that the protective effect of physical activity on frailty in people with a lower level of frailty is smaller. Another possible reason is that people with a high level of technology acceptance and literacy may have already engaged in more healthy behaviours [63]. Evidence showed that multiple strategies apart from physical activity also protect older adults from frailty (e.g., social and intellectual activities, dietary patterns) [64]. These protective associations may have dominated the associations of physical activity on frailty in the high technology acceptance group. This study, therefore, recommends that further studies should examine the possible negative dose-response relationship of physical activity on frailty in the population of older adults who are less frail, as well as the possible dominations of association by the protective strategies against frailty other than physical activity in the same population. This study also recommends in priority to target older adults who have a lower level of technology acceptance to engage in interventions promoting physical activities because this strategy may yield a larger protective effect against frailty.

This study has a number of limitations. First, the sample of this population is less frail, with a prevalence of frailty of 4.5%, compared to 9.9–13.6% globally, according to a systematic review.67 The findings of this study may only be applicable to a population of older adults who are less frail [2]. The associations observed in this study could only be transferrable to the population of older adults who are less frail. Second, the survey employed a self-filling online approach that recalls bias caused by possible dementia of the participants could be not excluded. Nevertheless, this sample was relatively young, with a mean age of 66.5 years. The prevalence of dementia in this young-old group (i.e., aged 67–69 years) was reported to be low (i.e., below 5%) [65]. Third, RAPA is a crude measure to quantify physical activity, albeit the fact that it has been validated. Further studies should confirm the findings of this study by using a more objective and accurate measurement (e.g., smartphone or accelerometer) [66]. Finally, the data collection period was conducted within the COVID-19 lockdown period. Physical activities which are usually conducted outdoors during the non-pandemic period were largely restricted. This may have limited the variability of the physical activity practised by older adults (i.e., shifted the amount to the lower side). It could have reduced the size of the effect of physical activity on frailty. It could have reduced the size of the effect of physical activity on frailty.

## Conclusion

This study showed that physical activity and technology acceptance were associated with frailty, and that technology acceptance moderated the association of physical activity with frailty. Future studies should examine the association of technology promotion with frailty in older adults. This study suggests that engaging older adults in physical activity should be prioritized in those with a low level of technology acceptance.

## Data Availability

The datasets generated and analyzed during the current study are not publicly available due to the restrictions involved when obtaining ethical approval for our study, which commit us to share the data only with members of the research team but allow data to be made available from the corresponding author upon reasonable request.

## Abbreviations

COVID-19: Coronavirus Disease of 2019
STROBE: Strengthening the Reporting of Observational Studies in Epidemiology
